# “Crying without tears” as an early diagnostic sign-post of triple A (Allgrove) syndrome: two case reports

**DOI:** 10.1186/s12887-017-0973-y

**Published:** 2018-01-15

**Authors:** Daniel Tibussek, Sujal Ghosh, Angela Huebner, Joerg Schaper, Ertan Mayatepek, Katrin Koehler

**Affiliations:** 1 Department of General Pediatrics, Neonatology and Pediatric Cardiology, University Children’s Hospital, Heinrich-Heine University, Moorenstrasse 5, 40225 Düsseldorf, Germany; 2 Department of Pediatric Oncology, Hematology and Clinical Immunology, Medical Faculty, Center of Child and Adolescent Health, Heinrich-Heine-University, Düsseldorf, Germany; 3 Department of Pediatrics, Medizinische Fakultät, Technische Universität Dresden, Dresden, Germany; 4 Department of Diagnostic and Interventional Radiology, Heinrich-Heine-University, Düsseldorf, Germany

**Keywords:** Allgrove syndrome, Achalasia, Alacrimia, Adrenal failure, Autonomic, Neuropathy, Triple A

## Abstract

**Background:**

Triple A syndrome (or Allgrove syndrome) is a rare autosomal recessive disorder characterized by alacrima, achalasia, adrenal insufficiency and autonomic/neurological abnormalities. The majority of cases are caused by mutations in the *AAAS* gene located on chromosome 12q13. However, the clinical picture as well as genetic testing may be complex since symptomatology is variable and mutations cannot be identified in all clinically diagnosed patients. We present two unrelated patients with triple-A syndrome illustrating the importance of alacrima as an early clinical sign.

**Case presentation:**

A 3.5 year old girl presented with repeated hypoglycaemic myoclonic events. Adrenal insufficiency was diagnosed. In addition, alacrima, obvious since early infancy, was incidentally reported by the mother and finally lead to the clinical diagnosis of triple A syndrome. This was confirmed by positive mutation analysis of the *AAAS* gene. The second patient, an 8 months old boy was presented because of anisocoria and unilateral optic atrophy. MRI revealed cerebellar vermis hypotrophy. Psychomotor retardation, failure to thrive, and frequent vomiting lead to further diagnostic work-up. Achalasia was diagnosed radiologically. In addition, the mother mentioned absence of tears since birth leading to the clinical diagnosis of triple A syndrome. In contrast to the first cases genetic testing was negative.

**Conclusion:**

These two patients illustrate the heterogeneity of triple A syndrome in both terms, clinical expression and genetic testing. We particularly aim to stress the importance of alacrima, which should be considered as a red flag symptom. Further differential diagnosis is required in every child affected by alacrima.

## Background

Triple A syndrome (OMIM#231550) is a rare autosomal recessive disorder [1]. Typically it is characterized by alacrima, corticotropin-resistent adrenal insufficiency and achalasia. The term 4A syndrome has been suggested [2] as additional autonomic dysfunctions may be associated [3, 4]. In addition, neurological abnormalities were frequently described [5]. Overlapping clinical signs with triple A syndrome shows the alacrima, achalasia, and mental retardation syndrome (AAMR, OMIM#615510). All AAMR cases described so far do not have adrenal insufficiency. The disorder is caused by mutations in guanosine diphosphate (GDP)-mannose pyrophosphorylase A (*GMPPA*) gene. Patients show furthermore delayed developmental milestones, hypotonia, gait abnormalities, anisocoria, and visual or hearing deficits [6].

Here we report on two children with this syndrome illustrating the variable clinical presentation and genetic heterogeneity. Furthermore, we like to stress the importance of alacrima as an important clinical sign in paediatric patients.

## Case presentation

### Case 1

This 3.5 year old girl is the first child of non-consanguineous parents of Moroccan origin.

During infancy she suffered from recurrent episodes of vomiting and regurgitation which later ceased without treatment. In addition, she was affected by frequent respiratory tract infections. She was referred to our clinic because of recurrent hypoglycaemic myoclonic events. The first episode occurred at the age of 2.5 years, while she was suffering from febrile gastroenteritis. After a fasting period of about 12 h the mother noted sudden generalized myoclonic jerks, which occurred about once a minute over a period of 30 min. The girl was fully responsive during that time. At the age of 3 years similar convulsions occurred during a fasting period related to a febrile respiratory infection. Oral application of a glucose solution given by her mother led to rapid cessation of the myoclonic jerks. Within the next months this observation recurred. An interictal EEG was found to be normal. Although not considered relevant by the mother she reported alacrima since early infancy. The child had been under ophthalmological supervision for keratoconjunctivits sicca since the age of 6 months.

Clinical examination revealed a dysplastic left auricle. No other dysmorphic features were found. Height and weight were within normal limits. Neurological examination was notable for symmetrically reduced deep tendon reflexes, spontaneous Babinski reflexes, and an increased muscle tone of the lower limbs. The gait was clumsy without other major signs of ataxia.

ACTH-resistant adrenal insufficiency was diagnosed by laboratory testing. Achalasia was excluded using a barium swallow test.

**Fig. 1:**
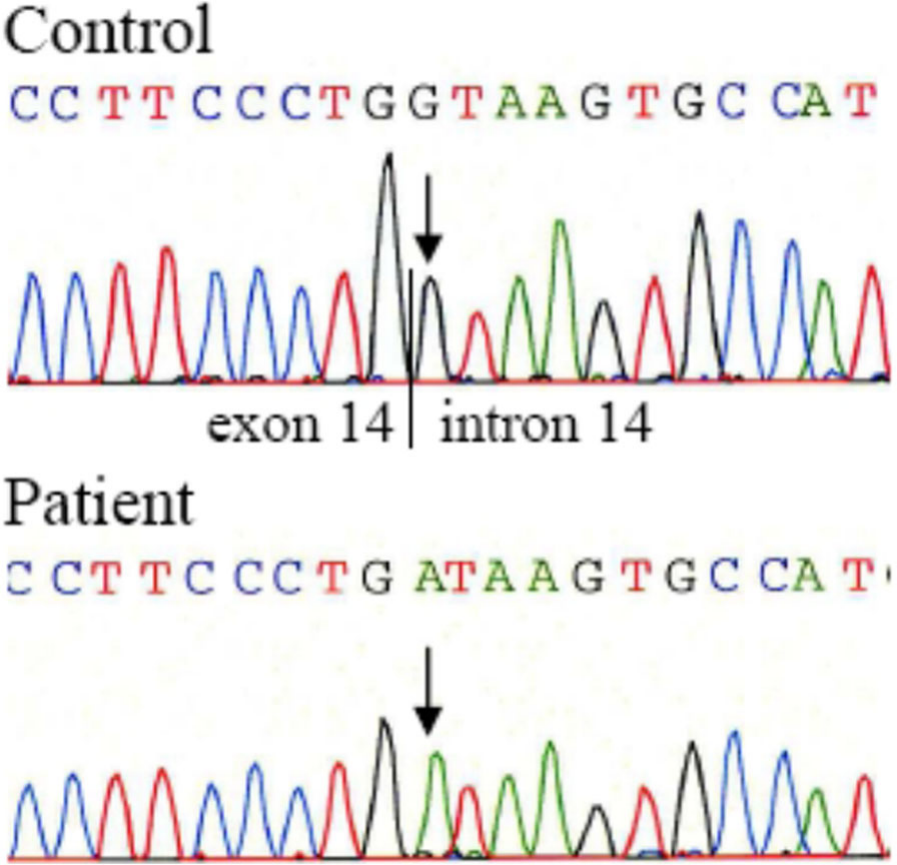
Sequence chromatograms of the patient and a control individual showing the G > A transition at nucleotide 1331 + 1 in intron 14 of the *AAAS* gene. The arrows indicate the nucleotide altered by mutation. The line indicates the exon-intron boundary

Mutation analysis showed a homozygous G > A transition at nucleotide position 1331 + 1 in intron 14 (c.1331 + 1G > A) resulting in a splice mutation at splice donor site of intron 14 (Fig. 1). This mutation results in a loss of an exon or inclusion of an intron and subsequently in aberrant splicing and a frameshift with a premature stop codon. The resulting truncated protein was most likely not functional.

### Case 2

**Fig. 2:**
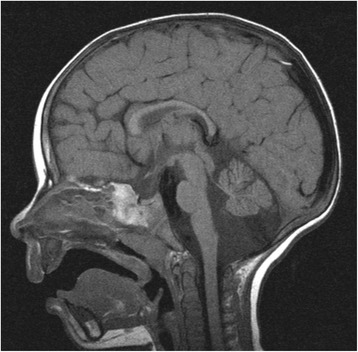
Cerebral MRI revealing an isolated hypotrophic caudal cerebellar vermis

**Fig. 3:**
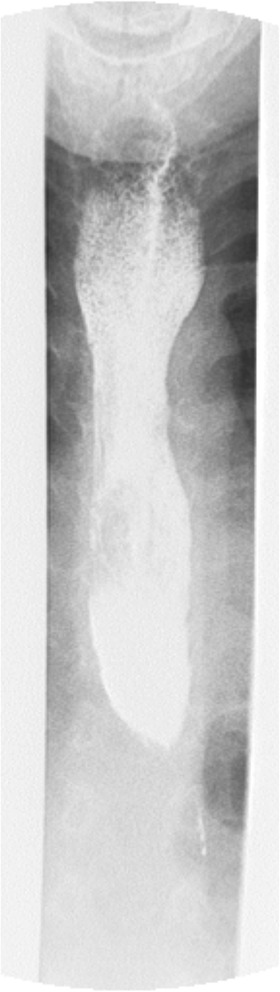
Barium swallow test of the patient demonstrated a dilated oesophagus and narrowing at the lower oesophageal sphincter confirming achalasia

This patient is the first child of first child of non-consanguineous parents. He presented at the age of 8 months because of anisocoria and reduced pupillary light response of the right eye. Ophthalmological examination revealed additional right optic nerve atrophy, myopia, and convergent strabism. Cerebral MRI showed isolated hypotrophic caudal cerebellar vermis (Fig. 2). At the age of 10 months the parents reported frequent vomiting after solid food and failure to thrive was noted (Weight < 3rd percentile). Progressive microcephaly was seen on follow-up. At the age of 1.5 years marked global developmental retardation became obvious. He was unable to walk or sit without support and did not exhibit any active speech. Hearing disorders were excluded by paedaudiological work-up. Further neurological examination revealed muscular hypotonia and week deep tendon reflexes. A comprehensive work-up for developmental delay, short stature and failure to thrive did not reveal any underlying pathology. In order to exclude neuroblastoma a chest x-ray was performed which revealed a broad vertical shadow in the mediastinum. Abdominal and thoracal ultrasound investigations were suspicious of achalasia, which was later confirmed by a barium swallow test (Fig. 3). At that time, the mother reported that since birth the child had never produced tears. Subsequently triple-A syndrome was suspected. No skin abnormalities or laboratory results suggestive of adrenal insufficiency were found so far.

Achalasia was surgically treated at the age of 20 months. On last follow-up at the age of 3 years, the patient was able to pull himself to stand and walked with assistance. Speech development was still severely delayed. His height and weight were still below the 3rd percentiles, although he showed mild catch-up growth. Mutation analysis of the coding sequence and all exon-intron junctions of the *AAAS* and *GMPPA* gene did not reveal a pathogenic mutation.

## Discussion

The two patients reported here emphasize that the triple A syndrome displays a phenotypic and genetic heterogeneity. In addition, the importance of alacrima as an important and early diagnostic sign is stressed is stressed.

In general, triple A syndrome can be considered as a multisystem disorder, which may be life-threatening when diagnosis is delayed. Clinically it is characterised by alacrima, usually present at birth, achalasia and adrenal insufficiency, which may only develop during childhood and adolescence. Many patients have associated neurological features [5] with involvement of the central, peripheral and autonomic nervous system.

In both of our patients alacrima, an autonomic feature, was the earliest clinical sign even leading to keratoconjunctivitis sicca in one. However, it was not considered relevant in the context of a possible underlying disease. Although alacrima is commonly described as the most consistent and early sign of triple A syndrome, in many patients alacrima has only been recognized as part of a congenital disorder in retrospect, after the occurrence of additional features of triple A syndrome. In both our patients alacrima had been observed by the patients’ mothers and patient 1 had additionally been under ophthalmological supervision due to “dry eye”. However, none of the patients had been referred for further differential diagnosis work-up.

Alacrima as a clinical sign is only seen in a limited number of congenital disorders, e.g. familial dysautonomia (hereditary sensory and autonomic neuropathy type III; HSAN3; MIM#223900), lacrimoauriculodentodigital syndrome (LADD; MIM#149730), the anhidrotic type of ectodermal dysplasia (MIM#224900), Sjögren syndrome (MIM#270150), congenital disorder of deglycosylation (MIM#615273), AAMR and triple A syndrome. Thus, it should always be taken serious if parents report on their children “crying without tears”. Careful history taking and clinical as well as laboratory examinations should usually lead to an underlying cause. Alacrima is very unlikely to be an isolated finding in children. This is particularly true if other autonomic features are present as seen in our second patient who exhibited anisocoria.

Further clinical symptoms in our patients were optic atrophy, achalasia and adrenal insufficiency. A high index of suspicion is warranted if “dry eyes” in children are associated with any of these clinical signs.

Achalasia, an unusual finding in children, may have a variable presentation in triple A patients. While some found achalasia as the first presenting symptom others found no evidence of achalasia at all [7]. Although we were not able to prove achalasia in patient 1, it may still occur later in childhood or even adolescence.

Anisocoria as another prominent clinical sign has been reported in up to 13% of previous triple A patients [8]. In general, 30% of triple A syndrome patients have been found to suffer from autonomic disturbances. These are predominantly parasympathetic [9].

The optic nerve atrophy seen in our patient as well as in previous cases [10] indicates a CNS involvement, specifically of white matter. Neurological abnormalities were present in both of our patients. Our first patient exhibited severe mental retardation and showed cerebellar vermal hypoplasia in the MRI, a finding that has so far not been described in the context of triple A syndrome. However, cerebellar ataxia was previously described in other patients. Patient 1 showed only mild global developmental delay. In addition, markedly reduced deep tendon reflexes were found probably indicating involvement of the peripheral nervous system. Peripheral polyneuropathy has been found as one of the frequent and progressive neurological findings in triple A patients [5, 11].

In patient 1 myoclonic episodes after longer periods of fasting were the main cause for referral. These were probably attributable to hypoglycaemia due to adrenal insufficiency since episodes could be terminated by oral glucose. Severe and even fatal hypoglycaemic episodes have been repeatedly mentioned as early signs of triple A syndrome. Importantly, adrenal insufficiency can still arise in late childhood [4] or even in adulthood [12]. Thus, early diagnosis of triple A syndrome is crucial in order to allow regular endrocrinological follow-up investigations and parent counselling to prevent symptomatic and potentially fatal hypoglycaemic episodes.

Although patient 2 showed typical clinical symptoms of triple A syndrome, he did not carry a pathogenic *AAAS* or *GMPPA* mutation. There could be two explanations for this. Firstly, there could be an unrecognised mutation, larger deletion or insertion in intronic sequences or the promotor of the *AAAS* or *GMPPA* gene. Or secondly, the patient suffers from a different triple A-like disease. In 2000 the triple A syndrome gene localized to chromosome 12q13 was cloned [13, 14]. The so called *AAAS* gene consists of 16 exons which encode a 546 amino acid protein called ALADIN (**Al**acrima-**A**chalasia-a**d**renal **I**nsufficiency **N**eurologic disorders). ALADIN is a protein of the nuclear pore complexes with a WD-repeat-domain structure [15]. Most of the mutant ALADIN proteins are mislocalized in the cytoplasm and are not available at all at the nuclear pore. However, the function of ALADIN at the nuclear pore is unknown. It is assumed that ALADIN plays an important role for protein and/or RNA trafficking between the nucleus and cytoplasm. It could be shown, that ALADIN deficiency impairs redox homeostasis in patient cells and inhibits steroidogenesis [16–18].

A number of genotype-phenotype studies have been undertaken underlining the marked phenotypic heterogeneity among affected patients even within one family [19, 20].

## Conclusion

Alacrima should be perceived as a red flag symptom in children and triple A syndrome should be considered if additional clinical features are present. Genetic testing should be taken into account even in the absence of all “classical” clinical signs. Our two patients illustrate the heterogeneity of triple A syndrome in terms of clinical expression and results of genetic testing.
